# Implementation of Paste Backfill Mining Technology in Chinese Coal Mines

**DOI:** 10.1155/2014/821025

**Published:** 2014-08-31

**Authors:** Qingliang Chang, Jianhang Chen, Huaqiang Zhou, Jianbiao Bai

**Affiliations:** ^1^School of Mines, Key Laboratory of Deep Coal Resource Mining, Ministry of Education of China, China University of Mining and Technology, Xuzhou 221116, China; ^2^School of Mines, China University of Mining and Technology, Xuzhou 221116, China; ^3^State Key Laboratory of Coal Resources and Safe Mining, China University of Mining and Technology, Xuzhou 221116, China

## Abstract

Implementation of clean mining technology at coal mines is crucial to protect the environment and maintain balance among energy resources, consumption, and ecology. After reviewing present coal clean mining technology, we introduce the technology principles and technological process of paste backfill mining in coal mines and discuss the components and features of backfill materials, the constitution of the backfill system, and the backfill process. Specific implementation of this technology and its application are analyzed for paste backfill mining in Daizhuang Coal Mine; a practical implementation shows that paste backfill mining can improve the safety and excavation rate of coal mining, which can effectively resolve surface subsidence problems caused by underground mining activities, by utilizing solid waste such as coal gangues as a resource. Therefore, paste backfill mining is an effective clean coal mining technology, which has widespread application.

## 1. Introduction

Clean mining is one kind of new production modes under strategy of sustainable development. After Environment and Development Conference of the United Nations proposed clean mining production in 1992, this type of production method has been gradually expanded within many countries and organizations in the world. In China, the government also actively expands the clean mining and put it into laws and regulations together with the ten 5-year project. The clean mining refers to applying the whole prevetion into production process and products and services, solving disadvantages of traditional end-of-pipe treatment. In this case, from the point of health economic criteria, the clean mining is just the “green mining.” The coal mining production requires that coal enterprises should concern the whole life cycle of coal products. The production safety processing control should be put into effect. From the perspective of mining design, processing and usage of coal resources, discharge of waste pollution should be controlled. This can reduce the undesirable impact on environments during the processing and transportation as well as usage of coal resources into minimum. In the same time, the combined usage of associated minerals should be considered, reducing the cost of resources as much as possible and acquiring the maximum economic profits [1].

Large-scale exploitation and utilization of coal in China provide guaranteed energy for industrial production and continued economic development. However, severe environmental pollution and ecological damage are also caused by mining activities [2]. At present, more than 95% of coal resources in China are obtained from underground coal mining. Coal mining activities ruin the landscape, leading to surface subsidence and decreasing the utilization ratio of land resources while discharged solid wastes such as gangues occupy and ruin the land [2]. Also, coal mining activities are harmful to water resources. Moreover, poisonous and harmful gases including SO_2_, CO_2_, and CO, produced by mining gangue autoignition, create air pollution [3]. To resolve these environmental problems induced by mining activities, numerous scientific and technical workers have studied and developed a series of advanced coal mining technologies through a long-term effort.

Domestic and international researchers together with technicists developed many technological measures to control the subsidence of overlying strata as well as ground. (1) Reserving protection pillars: this method is mainly used to protect the surface plant, shaft, and important constructions. What is more, it can be used in superficial part. Aiming at different protection objects, pillars with a certain size are reserved. After that, resources beyond the coal pillars can be mined and recycled. This kind of method is definitely the most effective when protecting surface buildings and constructions. However, this will lead to a serious waste of resources although those resources can be recovered when the mine is abandoned but with a low mining ratio. In the meantime, due to the specific characteristics of coal pillars, the layout difficulty of working faces is increased, restricting effective production. (2) Partly mining method: this mainly includes strip mining method, board and pillar, and mining with restricted thickness. With this method, under the condition that no wave mode subsidence occurs on the ground, 40% to 60% of coal resources can be dug out when appropriate ratio of mining and reserving is used. In America and South Africa [4], room and pillar mining method is nature. However, this method has some problems which are listed as follows. (a) A mass of resources were wasted and the recovery ratio is only limited between 40% and 60%. This ratio is even lower under conditions of thick seams and thin bedrock as well as deep mining. (b) Strip designed is relatively small. This is harmful for mechanized mining and brings more problems when the mining depth is large. (c) The mechanism of deep strip mining and subsidence is not fully understood. The traditional prediction model and method have a large difference with the practical values. (d) When multiple or thick coal seams are mined, coal pillars will deform and the strength will be reduced under the condition of external cause such as faults, creep effect, and mutation of tectonic stresses. This will increase the emergentness of subsidence damage, resulting in caving of roof with large area. (3) The backfill technology within the gap between gangues in the gob area means filling the gap between the gangues within the gob space using grouts before the gap is compressed fully. After the filled material glues the caved rock blocks, they will support the overlying strata together, making an effect to control the ground subsidence. This technology was initially used in Germany to fill the caved gob [5–9]. This can realise the parallel between mining and backfilling and have little effect on the production of working faces. Nevertheless, the problems are that the roof has already been damaged, caved, or subsided before backfilling. Thus, the backfilling effect cannot be promised [10]. (4) Grouting in the separation zone of overlying strata was initially proposed by the Soviet Union. The nature is that, after the coal seams are dug, the strata will curve and deform. When the shear stress between rock strata is beyond its maximum shear strength, there will be some relative movement between strata. If the stiffness of each stratum is different, the deflection deformation is not harmonious. There is isolation between strata and bed separation occurs. The grouting within the separation area is using cementitious grouts which will fill the separation space created during the movement process of overlying strata with the drilled holes between ground surface and separation space. In this way, the overlying strata deformation can be controlled, restricting surface subsidence. Countries like Poland started the field experimental research on overlying separation space. The controlling subsidence effect was introduced by Palaski [11]. This technology can reduce the deformation of ground surface to a value of 20% to 30%. In 1985, the Chinese researchers [12–14] introduced it into China. But there are still some problems here. For instance, the grout filling space is the bed separation area generated between strata, which is not the original source of surface subsidence or gob area. Thus, the overlying strata will also be compressed and subside, resulting in that it cannot meet the requirement of controlling ground subsidence. (5) Backfill mining: this mining method was started around the end of the 1990s. China began to apply it into industrial experiment in 1963 [15]. In the initial period, water and sands are always used in coal mines. But the problems are that firstly not only the transportation system of backfilling materials between the backfilling working face and ground surface should be established but also the drainage system needs to be built in the working face. With the working face advancing, the water retaining wall should be built to isolate the working face as well as the backfilling area. Furthermore, this kind of technology is outdated and sands are difficult to be obtained. Also, the mechanization of this mining method is difficult to be realised, which cannot meet the requirement of high yield and high active production.

In China, clean mining production of coal resources mainly includes aspects such as mining method, coal processing, and environment control. Within them, in the aspect of mining coal resources, kinds of clean mining technologies are applied, such as reducing discharging of gangues and coal gases development technology. Traditional caving method results in serious pollution to the environment around coal mines, mainly including discharging of coal gangues and kinds of gases. And the main reason for those problems is the excavated gob area.

New clean mining technology, techniques, and equipment are continuously being adopted by the mining industry, with the goal of minimizing the production of contaminants from the source or controlling the generation and pollution produced by coal mining activities [4]. The clean coal mining technology introduced in this paper is an example of a mining technology that can help control environmental pollution. Clean technology that is used in processing is not addressed here. Paste backfill mining technology, first proposed by China University of Mining and Technology, is regarded as an important component, having been successfully implemented in the first paste backfill mining demonstration experiment.

## 2. Concept and Principles of Paste Backfill Mining Technology

Due to different production requirement, in China, there are three types of backfilling methods, namely, high water material filling, gangue backfilling, and paste backfill mining. The high water material filling means two different materials, namely, A and B, are mixed together and transported to the gob area via pipelines. In the same time, the mixed liquid will cure to form the supporting body, resisting the overlying strata and realising the subsidence control. Among them, material A is mainly made up of aluminate or additives while material B consists of gypsum, lime, and clay. Both material A and material B are added into a certain amount of water to make grouts. After they are mixed with a ratio of 1 : 1, this grout will set within 30 minutes. However, after this kind of material cures, water may be easily lost, losing the supporting ability at the same time. Thus, this type of backfilling method can only be used in new mining area with full scale backfilling. Compared with that, gangue backfilling means that, after the coal gangues are broken, they are delivered to the working face via belt. Then, the waste gangues are transported to the scraper conveyer which is located under hydraulic backfilling support. After that, the gangues will be dropped via the discharging opening and be compressed via the tamping equipment, realising the green mining within the gob area. Although this kind of technology has the tamping organization, it is difficult to handle and the subsidence controlling effect is relatively worse. Thus, this type of backfilling method can only be used when there is no rigid requirement.

**Figure 1:**
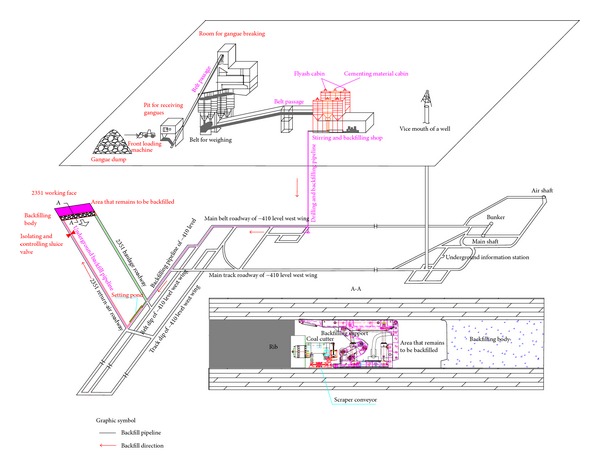
Diagram of paste backfill technology.

Paste backfill mining (see Figure 1) is a coal mining method in which solid mining wastes such as gangues are broken and processed and then mixed with flyash, cementing materials, and water in a specific proportional basis. After that, the mixture is stirred, becoming coagulable paste slurry. The paste slurry is then transported to the paste backfilling face through pipelines via pump pressure and gravity, filling the full gob timely and effectively. On one hand, this method can protect surface buildings (e.g., village homes); on the other hand, it can improve the safety of coal mining and recovery rate of coal resources. This method also utilizes the paste waste such as gangues and flyash [4]. Among them, coal ash is mainly from thermal power plant. The main composition elements are SiO_2_, Al_2_O_3_, FeO, Fe_2_O_3_, CaO, TiO_2_, MgO, K_2_O, Na_2_O, SO_3_, MnO_2_, and so forth. Coal gangues are the rock blocks during coal production or underground excavation in roadways. Cementing materials are mainly divided into two types: SL material is produced based on industry wasters such as furnace slag and steel slag (moderate natural minerals and cinder are mixed together to make the new grouting material which can be used in mines), while PL material is mixed with normal cement and natural resources as well as composite additives, which is appropriate for backfill mining in underground mines.

In paste backfill mining, because drilling is above ground and paste backfill materials are transported and mixed with the gob through pipelines, surface subsidence of the overlying strata is controlled. The technology is based on the theory of the balance model of mining overlying a strata beam. In this case, on the top of the gob, a stress balance arch is formed. The self-weight of the strata causes the arch to bend and subside. With backfilling, the overlying strata above the gob gradually become the overlying strata beam owing to the support of the paste backfill body. After the paste backfill body is compressed, it achieves a triaxial stress state with respect to its load-carrying capability. In the finite compressible range of the paste backfill body, the overlying strata beam deforms identically to the compressing paste backfill body. Afterward, the paste backfill body converts to a part of the rock mass, supporting the overlying strata and preventing them from deforming or being damaged. Ultimately, the goal of controlling surface subsidence is realized [16].

## 3. Characteristics and Process of Paste Backfill Mining Technology

Paste backfill mining offers a novel approach for supporting the overlying strata and controlling surface subsidence. With regard to the coal mining technology of “Under Three Objects,” combined with the paste backfill practice which has been done by the research group as well as the good results obtained from that practice, it can be concluded that paste backfill technology fulfills the technology requirement that coal mine technology should be in accordance with a sustainable development policy, reducing environmental pollution and improving recovery rates of resources. Moreover, paste backfill mining technology reduces pollution in the mining environment in a number of ways [17].

1. The mining technology reduces surface subsidence after the coal is mined. Therefore, the effect that mining activities have on the surface environment decreases. Because the solid waste is used to backfill, this resolves the problem of land being occupied by the solid waste, reducing the damage to the landscape.
2. Implementation of this mining technology can improve underground working conditions, by effectively preventing water damage, gas outburst, and rock burst.
3. This technology can increase the resource recovery rate and improve the level of clean mining. It can reduce the amount of damage to surface constructions from mining activities, making mining more socially acceptable to local residents.
4. It can reduce the amount or time needed for mining and shorten the time period in which mining impacts the surface environment, decreasing environmental disruption.

**Figure 2:**
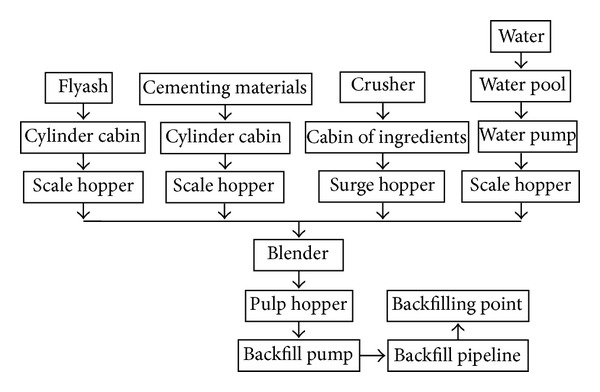
The paste backfill technological process.

This technology does not need to resort to underground measures and can be implemented without changing the presently developed mining system. Managing the mining operation is combined with environmental protection. The technology can help control strata movement and surface subsidence, providing underground storage for flyash generated in electric power plants and gangues produced in coal mines. Therefore, it is beneficial for protecting the environment and reducing the loss of underground water. Furthermore, the range of application is wide. Not only can this technology be used by itself but it can also be combined with other measures. Its technological process is shown in Figure 2.

## 4. Constitution and Characteristics of Paste Backfill Materials

To reduce the cost of backfilling and adapt to the diversity of material sources, our research group studied and developed specified backfilling cementing materials such as PL and SL that have features of early strength and rapid setting. It should be mentioned that the early strength means the strength of material after it sets for 8 h while the long-term strength equals the strength after the material cures for 28 days. For the same mass concentration (80%), we compared experimental results of PL backfilling cementing materials and 42.5 level normal cement, which agglutinates different backfilling materials. Our results are listed in Table 1.

According to Table 1, compared with cement, our specified cementing material has advantages of low production cost, less dosage, rapid setting, and high strength. Furthermore, the paste backfilling aggregate can be solid waste (e.g., gangue), fine sand, loess, or municipal solid waste. Under usual conditions, the paste backfill slurry has the following features [18].

1. It is highly concentrated. The slurry does not precipitate nor bleed. Moreover, the slurry does not liquefy either. Normally, the mass concentration of paste backfilling slurry is >75% and the maximum concentration reaches 88%, whereas the normal concentration of materials used in sand filling is <65%. Therefore, the backfilling face does not need complicated drainage equipment, avoiding or reducing the effect filling water has on the working face. Furthermore, the backfilling compaction rate is high.
2. The flow condition is plunger structure flow, which does not have critical velocity. The paste backfilling slurry essentially slides horizontally in the pipeline. On the whole, the slurry throughout the cross section of the pipeline flows at the same speed. Gangues used in paste backfill mining only need to be broken and processed, which can reduce the cost of processing materials. In addition, such low-velocity transport can reduce pipeline attrition.
3. The strength is higher under identical cementing material dosage conditions. Therefore, one can reduce the dosage of cementing materials that are more expensive, reducing material cost.
4. The compression ratio of the backfill body is lower, being ~1%. The compression ratio of normal sand fill (including artificial sands) is around 10% and the grading difference reaches nearly 20%. The interspaces among solid particles in the paste backfill materials are filled with cementing materials and water.

## 5. System Composition and the Technical Process of Backfilling

The paste backfilling system for the Daizhuang Coal Mine comprises five sections; all of them are with automatic centralized control systems in place. The system composition and major technical functions are as follows.

1. Gangue breaking and processing system: loading machines are used to put the gangues into the feeding hopper of original gangues. After that, the gangues are transported to a vibrating screen and loaded into the crusher as well as the feeding hopper of final gangues, which are prepared for paste backfilling usage.
2. Matching and stirring system: each constituent part of the paste backfill slurry is stirred in a blender until the stated time according to the given proportion. Then the slurry is unloaded into the slurry surge hopper for pumping.
3. Pipeline pumping system: the slurry in the surge hopper enters the backfilling pump cavity, relying on self-gravity. After being forced by the backfilling pump, the slurry reaches the backfilling face by means of a pipeline, putting the sequence backfilling of the gob into effect.
4. Control system: monitoring is performed on the material location, water, weight, quantity of flow, and pressure of the backfilling pump.
5. Dedusting system: dedusting equipment is arranged in segments for extracting, stirring, material loading, storage, and preventing environmental and noise pollution.

In the fully mechanized paste backfill technical process, the gangues are first broken and processed. Then four kinds of materials—gangues, flyash, specified cementing materials, and water—are proportionally mixed and stirred to make the paste slurry. After that, the paste slurry is transferred to the underground paste backfilling face by means of the backfilling pump. When the back mining is finished, hydraulic supports combined with auxiliary isolated measures form the closed gob space.

Therefore, coal cutting is conducted according to a mechanized technique. The backfilling step is 3 m and the narrow web is 0.8 m. After the coal cutter cuts four knives, mechanized paste backfilling can be conducted. The major technical process is as follows.

1. Backfilling preparation: the backfilling area is first sealed and isolated. The backfilling pipelines are connected and fixed.
2. Checking preparation: the on-off state of the backfilling pipe switches is checked and a report is sent to the backfilling stations, confirming whether or not the area should be backfilled.
3. Paste backfilling: this stage includes pipeline water filling, driving water by grouts, driving grouts by gangue slurry, and normal backfill.
4. Driving gangue slurry by mortar: a few grouts are allocated. It is then confirmed that clean water cannot mix with gangue slurry when cleaning the pipeline.
5. Driving slurry by water: clean water is put into the slurry hopper. This cleaning water is discharged into the drainage roadway by means of drainage pipelines.
6. Driving water by air pressure to clean the pipeline: pressurized air is used to blow out the clean water and other remaining particles in the pipeline, accomplishing the work of pipeline cleaning.

## 6. Analysis of the Engineering

**Figure 3:**
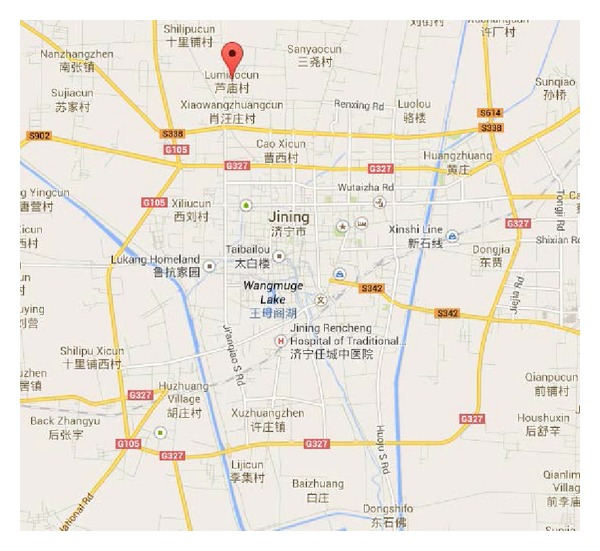
Location of Daizhuang Coal Mine in Jining.

Daizhuang Coal Mine, as shown in Figure 3, which belongs to Shandong Zibo Vinacomin, is located to the north of Jining City, Shandong Province. During the initial stage of coal mining here, mining coal under buildings posed a problem. Therefore, strip mining was adopted. Strip mining always produces a mass of coal pillars, leading to low recovery rate and a waste of valuable coal resources. This is not beneficial to sustainable development of mines and cannot meet the requirement of surface subsidence protection as urbanization of villages increases and the expansion of cities speeds up.

In 2008, Daizhuang Coal Mine in conjunction with China University of Mining and Technology as well as Xuzhou CUMT Backfill Technology Limited Corporation developed the research of mechanized paste backfill technology. The paste backfill working face is laid out in the west of the coal mine where the coal seam thickness is 2.65 m. The mining depth is 450 m and the dip angle of the coal seam is 3°–8°, with an average of 5°. The lithology of the roof above the coal seam is relatively hard and the integrity is good, indicating that it is a mediumly stable roof. The average thickness of the roof is 6.95 m. The length of the working face is 102 m and the strike length is 1150 m. The protected objects on the working face include Tianjutang Factory, Jin Village, Shili Store, and National Road 105 that crosses from the northeast direction on the working face. The method of paste backfill mining with the full gob being backfilled was adopted to control the roof.

Then the scheme was demonstrated, with civil design and construction being conducted first. After the equipment was installed and debugged, the first successful experimental practice of paste backfill technology at Daizhuang was demonstrated in December 2009. During the engineering, the tail beam of the hydraulic support effectively controlled the roof-to-floor convergence before backfilling, while the density of head support increased. Moreover, the specified backfilling support was combined with magnetic foam plastics and mat patch, guaranteeing complete sealing of the delaying backfilling area. The content of gangues existing in the backfilling materials was improved, reducing the compression ratio of the backfilling body.

**Figure 4:**
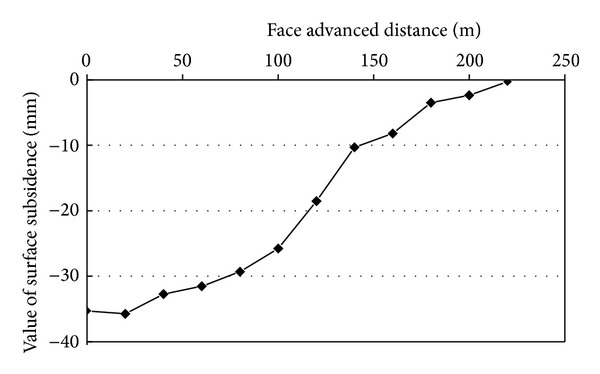
A practical measuring curve of the surface subsidence induced by paste backfill mining.

By February 2011, 400 m of footage had been accomplished. The recovery rate of coal resources improved from the original 45% generated from strip mining to 95%. The maximum subsidence was 35 mm, as shown in Figure 4. The total backfilling reached >0.12 million and the backfilling ratio was >97%. The coal resources taken out using paste backfilling amounted to 0.17 million tons. The direct economic benefit was 0.86 billion yuan. The amount of gangue consumed was 108,000 tons and the amount of flyash consumed was 48,000 tons. The amount of water consumed was 36 km^3^.

This demonstration shows that not only can paste backfill technology improve the excavation rate but it can also extend the service life of coal mines, while effectively controlling mining subsidence. Furthermore, this technology can ease the tension between coal workers and local residents, realizing more sustainable coal mining. In addition, by making use of solid waste as a resource, this technology achieves a cleaner way of mining coal resources.

This technology can be applied to many technical areas such as surface control, mining with water protection, hard roof control, gob-side entry retention, and disposal of solid waste. Furthermore, it has been applied to similar coal mines such as Jiaozuo, Fengfeng, Zibo, and Hebi collieries.

## 7. Conclusions

With the rapid development of China's economy, development and utilization of coal resources will increase. Thus, more attention must be paid to the induced waste of resources and environmental pollution. To resolve these problems, coal enterprises must implement cleaner mining technologies. Coal mines should employ corresponding clean mining technology during the production process, improving the safety and recovery rate of coal mining. At the same time, pollution sources should be effectively controlled to achieve long-term, stable, safe, and environmental sustainable development.

In this paper, we give a relatively systemic and comprehensive introduction to paste backfill technology, interpreting the technical mechanism and process of paste backfill technology. Also, the constitution and features of the backfilling materials, the composition of the backfilling system, and the technical process are given. Combined with engineering practice, this technology can improve the recovery rate and help control surface subsidence. Our results show that the recovery rate obtained by using paste backfill technology can reach >95% and that surface buildings can be controlled in the I damaging scope.

Not only can this technology improve the recovery rate of coal resources to fulfill the policy of resource-saving technical development but it also is beneficial to solid waste management. Therefore, it has an extremely strategic importance in developing coal resources in China, producing clean energy, ensuring a secure energy supply for China, improving China's world competitiveness, and promoting economic development while addressing environmental concerns.

## Figures and Tables

**Table 1:** Performance table of PL backfilling cementing materials and 42.5 level normal cement.

Kind of aggregate	Cementing materials (kg*·*m^−3^)		Compressive strength (MPa)				
	Variety	Dosage	8 h	1 d	3 d	7 d	28 d
Fine sand from Si River	PL	80	0.16	0.70	1.45	1.88	2.43
		100	0.31	1.00	2.13	2.71	3.38
		150	0.63	1.54	3.25	4.33	5.47
		250	1.22	2.07	6.22	8.34	10.2
	Cement	100	0	0.29	0.63	0.92	2.13

Loess from Huozhou	PL	80	0	0.18	0.30	0.38	0.51
		100	0	0.36	0.47	0.63	0.79
		120	0.22	0.47	0.71	0.85	1.10
		150	0.41	0.66	0.91	1.15	1.47
	Cement	100	0	0	0.16	0.31	0.72
		120	0	0	0.20	0.36	1.07
		150	0	0	0.31	0.58	1.58

Gangue	PL	200		0.54	1.73	3.74	6.40
		250		1.58	3.79	5.20	7.24
		300		2.90	5.28	6.62	9.94
	Cement	200		0.51	0.84	1.04	1.63
		250		0.58	1.29	1.49	2.45
		300		0.66	1.71	2.16	3.61
